# Sex Differences in the Neuropsychiatric Symptoms of Multiple Sclerosis: Symptômes neuropsychiatriques de la sclérose en plaques: différences selon le sexe

**DOI:** 10.1177/07067437261425067

**Published:** 2026-02-28

**Authors:** David E. Freedman, Jiwon Oh, Gillian Einstein, Anthony Feinstein

**Affiliations:** 1 Department of Psychiatry, 71545 Sunnybrook Health Sciences Centre, Toronto, ON, Canada; 2 Department of Psychiatry, Temerty Faculty of Medicine, University of Toronto, Toronto, ON, Canada; 3 Division of Neurology, Department of Medicine, 10071 St. Michael's Hospital, Toronto, ON, Canada; 4 Division of Neurology, Temerty Faculty of Medicine, University of Toronto, Toronto, ON, Canada; 5 Department of Psychology, 7938 University of Toronto, Toronto, ON, Canada; 6 7938 BARLO MS Centre, St. Michael's Hospital, Toronto, ON, Canada

**Keywords:** Sex, depressive disorders, anxiety, cognitive deficits, fatigue, multiple sclerosis

## Abstract

**Objective:**

There are clinical and radiological sex differences in people with multiple sclerosis (pwMS); however, the influence of sex on the neuropsychiatric symptoms of MS remains unclear. This study aimed to clarify whether there are sex differences in the neuropsychiatric symptoms of MS.

**Method:**

A consecutive clinical cohort of 1530 pwMS completed the Hospital Anxiety and Depression Scale for symptoms of anxiety (HADS-A) and depression (HADS-D), the Modified Fatigue Impact Scale (MFIS), the Perceived Deficits Questionnaire (PDQ) for subjective cognitive concerns, and the Minimal Assessment of Cognitive Function in MS neurocognitive battery. Linear regression analyses were undertaken to predict neuropsychiatric raw scores from sex, adjusting for age, educational years, Expanded Disability Status Scale (EDSS) scores, disease duration, and disease subtype. The significance threshold was set at *p* < .01.

**Results:**

Mean age was 43.2 years (SD = 10.6), mean disease duration was 9.7 years (SD = 8.4), and median EDSS was 2.0 (IQR = 1.5–3.5). Seventy-three percent of participants were female. Controlling for covariates, females had higher MFIS, PDQ, California Verbal Learning Test, and Brief Visuospatial Memory Test total learning scores, and lower Judgment of Line Orientation and Paced Auditory Serial Addition Test scores than males, all *p* < .01. Sex was not independently predictive of scores on the HADS-A, HADS-D, Controlled Oral Word Association Test, Brief Visuospatial Memory Test delayed recall, Symbol Digit Modalities Test, or Delis-Kaplan Executive Function System.

**Conclusions:**

Among pwMS, females experience greater fatigue, cognitive complaints, and dysfunction in spatial rotation and working memory than males, while males have elevated learning/memory difficulties. The absence of differences in symptoms of depression and anxiety between males and females contrasts with well-described sex differences in the general population. These data support future research that can explain sex-specific mechanisms of neuropsychiatric symptoms in pwMS.

## Résumé

**Objectif:**

Des différences cliniques et radiologiques selon le sexe ont été observées chez les personnes atteintes de sclérose en plaques (SEP). Toutefois, l’influence du sexe sur les symptômes neuropsychiatriques de la SEP demeure non élucidée. La présente étude visait à clarifier s’il existe des différences selon le sexe dans ces symptômes.

**Méthode:**

Une cohorte clinique consécutive de 1 530 personnes atteintes de SEP a été évaluée à l’aide de l’*Échelle d’anxiété et de dépression en milieu hospitalier* pour les symptômes d’anxiété (HADS-A) et de dépression (HADS-D), de l’échelle d’impact sur la fatigue modifiée (MFIS), du *Perceived Deficits Questionnaire* (PDQ) pour les plaintes cognitives subjectives et des tests neurocognitifs d’évaluation minimale de la fonction cognitive dans le contexte de la SEP. Des analyses de régression linéaire ont été réalisées afin de prédire les scores bruts des mesures neuropsychiatriques à partir du sexe, ajustés selon l’âge, le nombre d’années de scolarité, le score à l’échelle étendue du statut d’invalidité (EDSS), la durée de la maladie et le sous-type de maladie. Le seuil de signification a été fixé à *p* < 0,01.

**Résultats:**

L’âge moyen des participants était de 43,2 ans (écart-type = 10,6), la durée moyenne de la maladie de 9,7 ans (écart-type = 8,4) et le score médian à l’EDSS de 2,0 (écart interquartile = 1,5; 3,5). Soixante-treize pour cent des participants étaient des femmes. Après ajustement pour les covariables, les femmes présentaient des scores plus élevés à la MFIS et au PDQ, ainsi que des scores d’apprentissage total supérieurs au *California Verbal Learning Test* et au *Brief Visuospatial Memory Test*, mais des scores plus faibles au *Judgment of Line Orientation* et au *Paced Auditory Serial Addition Test* que les hommes (tous *p* < 0,01). Le sexe n’était pas un facteur prédictif indépendant des scores à la HADS-A, à la HADS-D, au *Controlled Oral Word Association Test*, au rappel différé du *Brief Visuospatial Memory Test*, au *Symbol Digit Modalities Test* ni au *Delis-Kaplan Executive Function System*.

**Conclusions:**

Chez les personnes atteintes de SEP, les femmes présentent davantage de fatigue, de plaintes cognitives et d’altération des capacités de rotation spatiale et de mémoire de travail que les hommes, tandis que ces derniers affichent davantage de difficultés d’apprentissage et de mémoire. L’absence de différences liées au sexe pour les symptômes de dépression et d’anxiété contraste avec celles, bien établies, qui sont observées dans la population générale. Ces résultats soutiennent la poursuite de recherches visant à mieux comprendre les mécanismes sexospécifiques sous-jacents aux symptômes neuropsychiatriques de la SEP.

## Introduction

Multiple sclerosis (MS) is an immune-mediated demyelinating condition with sex differences in MS susceptibility, disease activity, and radiological abnormalities.^1,2^ While females are at higher risk of developing MS than males, males are more likely to experience a progressive illness and demonstrate greater whole brain and regional gray matter atrophy.^1,2^ These differences may be due to sex chromosomes, sex hormones, sociocultural factors, or combinations thereof. As such, characterizing the differences between males and females can reveal novel avenues for research to improve care for pwMS. Yet, the evidence is equivocal about how sex influences the neuropsychiatric symptoms of MS.

People with MS (PwMS) are burdened by diverse neuropsychiatric sequelae, such as depression, anxiety, fatigue, subjective cognitive concerns, and objective cognitive dysfunction.^3^ These symptoms influence the quality of life and functioning of pwMS.^3^ However, there is uncertainty about whether there are neuropsychiatric sex differences in pwMS. Some studies suggest elevated anxiety in females,^4,5^ while others do not.^6,7^ Most research does not support a sex difference in depressive symptoms,^6,7^ but a few recent studies differ.^4,8,9^ While studies generally demonstrate higher fatigue in females than males,^10,11^ one investigation indicates more severe fatigue in males.^12^ There is limited evidence about sex differences in subjective cognitive concerns.^8,13^ In addition, while several studies *report* broadly increased cognitive dysfunction in males with MS,^1,12^ the existing data are highly variable. Although studies about cognition in pwMS generally show stronger verbal learning/memory in females compared to males,^6,8,14,15^ there is mixed data about sex differences in visual learning/memory,^6,8,14–16^ processing speed,^2,6,8,14–17^ and working memory.^6,8,14,15^ Few studies have examined executive function and spatial processing.^6,15^

Some of the ambiguity about neuropsychiatric sex differences in pwMS may be attributed to small sample sizes,^2,6,14–16^ failures to account for potential confounding factors,^5,11–13^^,17^ or the use of diagnostic data rather than direct symptom measures.^4,7^ Furthermore, pwMS often experience multiple overlapping neuropsychiatric symptoms, but this landscape is not captured by studies that only examine sex differences in one,^5,8–12^^,14–17^ two,^4,7^ or three symptoms at a time.^6,8^ Examining symptoms in isolation may fragment our understanding of the overall experience of pwMS. What is therefore needed to avoid perpetuating the problem is a robust study comparing the sexes across a comprehensive array of symptoms elicited with validated psychometric measures. This forms the focus of our study.

## Methods

Data were extracted from the clinical records of a consecutive cohort of 1530 people aged 18–65 with MS (diagnosed per the McDonald criteria)^18^ seen at a tertiary neuropsychiatry clinic in Toronto, Canada from 2006 to 2025. Participants completed neuropsychological testing during routine clinical care.

Demographic and disease-related data included sex (stratified into males and females), age, years of education, disease duration (in years), disease subtype, disease-modifying therapy use, and neurological disability, assessed with the Expanded Disability Status Scale (EDSS).^19^

Cognition was evaluated with the Minimal Assessment of Cognitive Function in MS (MACFIMS) neurocognitive battery.^20,21^ The MACFIMS battery includes tests of verbal fluency (Controlled Oral Word Association Test; COWAT), visuospatial processing (Judgment of Line Orientation test; JOLO), verbal learning and memory (California Verbal Learning Test Second Edition; CVLT), visuospatial learning and memory (Brief Visuospatial Memory Test – Revised; BVMT), processing speed (Symbol Digit Modalities Test; SDMT), working memory (Paced Auditory Serial Addition Test; PASAT), and executive function (Delis-Kaplan Executive Function System; D-KEFS), The CVLT and BVMT include scores for total learning (CVLT_TL and BVMT_TL) and memory (CVLT_DR and BVMT_DR), the PASAT has scores for three-second (PASAT_3sec) and two-second versions (PASAT_2sec), and the D-KEFS has scores for correct sorts (D-KEFS_CS) and description (D-KEFS_DS).^20^ Symptoms of anxiety and depression were assessed with the Hospital Anxiety and Depression Scale sub-scales for anxiety (HADS-A) and depression (HADS-D) respectively.^22^ Fatigue was measured with the Modified Fatigue Impact Scale (MFIS) and subjective cognitive concerns were measured with Perceived Deficits Questionnaire (PDQ).^23,24^ Decreased scores on the MACFIMS, and elevated HADS-A, HADS-D, MFIS, and PDQ scores, all validated measures in pwMS, indicate increased dysfunction. Collection of MFIS and PDQ data began in 2018 (with recent discontinuation) and 2020 respectively, contributing to less data for these measures.

In preliminary testing, *t*-tests (for continuous variables) or chi-square analyses (for categorical variables) were conducted to compare demographic, disease-related, and neuropsychiatric data between males and females. We calculated overlap coefficients to estimate the level of similarity in neuropsychiatric data between males and females. Linear regression analyses were conducted to predict raw scores on the HADS-A, HADS-D, MFIS, PDQ, and MACFIMS from sex, adjusted for age, years of education, EDSS scores, disease duration, and disease subtype (stratified into relapsing or progressive illness). The threshold for statistical significance was set at *p* < .01 to account for multiple statistical tests. Missing data were addressed by listwise deletion in each linear regression analysis.

## Results

As described in Table 1, the mean participant age was 43.18 years (SD = 10.58), the average years of education was 15.79 years (SD = 2.95), and the mean disease duration was 9.67 years (SD = 8.38). Most participants had a mild-moderate level of disability, 83.26% had relapsing-remitting MS, and 71.02% were receiving a disease-modifying therapy. Seventy-three percent of the participants were female.

**Table 1.** Sample Descriptive Data (n = 1530).

	Number of Participants	Mean	Standard deviation
Age (years)	1530	43.18	10.58
Years of education	1529	15.79	2.95
Expanded disability status scale (EDSS)	1484	2.61	1.81
EDSS (median and interquartile range)	1484	2.00	1.50–3.50
Disease duration (years)	1231	9.67	8.38
		Percentage	
Female sex	1120/1530	73.20	
Disease subtype			
Relapsing-remitting MS	1253/1505	83.26	
Secondary progressive MS	143/1505	9.50	
Primary progressive MS	109/1505	7.24	
Any disease-modifying therapy	1071/1508	71.02	
		Mean	Standard deviation
HADS-A	1496	9.54	4.36
HADS-D	1496	6.96	4.02
MFIS	886	49.61	17.10
PDQ	973	39.85	15.66
MACFIMS raw scores			
COWAT	1493	36.06	11.76
JOLO	1482	23.74	4.67
CVLT_TL	1517	50.98	12.44
CVLT_DR	1515	10.78	3.96
BVMT_TL	1485	21.54	7.65
BVMT_DR	1484	8.26	3.02
SDMT	1406	47.59	13.08
PASAT_3sec	1423	39.76	12.46
PASAT_2sec	1302	30.48	9.98
D-KEFS_CS	1492	9.43	2.77
D-KEFS_DS	1492	34.80	11.12

*Note:* MS = multiple sclerosis; HADS-A = Hospital Anxiety and Depression Scale sub-scale for symptoms of anxiety; HADS-D = Hospital Anxiety and Depression Scale sub-scale for symptoms of depression; MFIS = Modified Fatigue Impact Scale; PDQ = Perceived Deficits Questionnaire; MACFIMS = Minimal Assessment of Cognitive Function in Multiple Sclerosis; COWAT = Controlled Oral Word Association Test; JOLO = Judgment of Line Orientation; CVLT_TL = California Verbal Learning Test Second Edition Total Learning score; CVLT_DR = California Verbal Learning Test Second Edition Delayed Recall score; BVMT_TL = Brief Visuospatial Memory Test Revised Total Learning score; BVMT_DR = Brief Visuospatial Memory Test Revised Delayed Recall score; SDMT = Symbol Digit Modalities Test; PASAT_3sec = Paced Auditory Serial Addition Test 3-s version; PASAT_2sec = Paced Auditory Serial Addition Test 2-s version; D-KEFS_CS = Delis-Kaplan Executive Function System Correct Sorts score; D-KEFS_DS = Delis-Kaplan Executive Function System Descriptive score.

Sex-stratified data are included in Table 2. Female pwMS had longer disease duration (*p* < .01), lower neurological disability (*p* < .01), and were more likely to have relapsing-remitting MS (*p* < .01) compared to male pwMS. There were no sex differences in age, years of education, or use of disease-modifying therapy.

**Table 2.** Sample Data Stratified by Sex.

	Males (*n* = 410)		Females (*n* = 1120)				
	Mean	SD	Mean	SD	*df*	*t*	
Age (years)	43.32	10.33	43.13	10.67	1528	0.31	
Education (years)	15.49	3.13	15.90	2.88	680^a^	−2.32	
Expanded disability status scale	2.86	2.01	2.52	1.73	622^a^	3.18**	
Disease duration (years)	8.40	7.29	10.11	8.68	653^a^	−3.43**	
	*n*	%	*n*	%	*df*	*X* ^2^	
Disease course (RMS vs. PMS)					1	20.31**	
RRMS	305/401	76.06	948/1104	85.87			
SPMS	38/401	9.48	105/1104	9.51			
PPMS	58/401	14.46	51/1104	4.62			
Any disease-modifying therapy	290/403	71.96	781/1105	70.68		0.24	
	Mean	SD	Mean	SD	*df*	*t*	Overlap coefficient
HADS-A	8.92	4.51	9.76	4.28	1494	3.32**	0.99
HADS-D	7.05	4.12	6.93	3.98	1494	0.52	1.00
MFIS	47.17	16.72	50.35	17.16	884	2.34	0.98
PDQ	37.07	16.04	40.75	15.45	971	3.16**	1.00
COWAT	35.27	11.78	36.34	11.74	1491	1.56	0.97
JOLO	25.34	4.42	23.16	4.62	1480	8.14**	0.94
CVLT_TL	45.96	11.97	52.81	12.11	1515	9.78**	0.98
CVLT_DR	9.45	3.91	11.27	3.87	1513	8.06**	1.00
BVMT_TL	20.33	7.81	21.97	7.54	1483	3.66**	0.99
BVMT_DR	7.92	3.17	8.38	2.96	1482	2.61**	0.99
SDMT	46.05	13.07	48.14	13.05	1404	2.65**	0.99
PASAT_3sec	41.69	12.72	39.05	12.30	1421	3.58**	1.00
PASAT_2sec	32.08	10.52	29.87	9.71	606^a^	3.46**	0.99
D-KEFS_CS	9.65	2.83	9.35	2.74	1490	1.87	1.00
D-KEFS_DS	35.23	11.65	34.64	10.92	1490	0.90	0.99

*Note:* SD = standard deviation; df = degrees of freedom; MS = multiple sclerosis; RRMS = Relapsing-Remitting MS; PMS = progressive MS; SPMS = secondary PMS; PPMS = primary PMS; HADS-A = Hospital Anxiety and Depression Scale sub-scale for symptoms of anxiety; HADS-D= Hospital Anxiety and Depression Scale sub-scale for symptoms of depression; MFIS = Modified Fatigue Impact Scale; PDQ = Perceived Deficits Questionnaire; MACFIMS = Minimal Assessment of Cognitive Function in Multiple Sclerosis; COWAT = Controlled Oral Word Association Test; JOLO = Judgment of Line Orientation; CVLT_TL = California Verbal Learning Test Second Edition Total Learning score; CVLT_DR = California Verbal Learning Test Second Edition Delayed Recall score; BVMT_TL = Brief Visuospatial Memory Test Revised Total Learning score; BVMT_DR = Brief Visuospatial Memory Test Revised Delayed Recall score; SDMT = Symbol Digit Modalities Test; PASAT_3sec = Paced Auditory Serial Addition Test 3-s version; PASAT_2sec = Paced Auditory Serial Addition Test 2-s version; D-KEFS_CS = Delis-Kaplan Executive Function System Correct Sorts Score; D-KEFS_DS = Delis-Kaplan Executive Function System Descriptive Score.

^a^Significant Levene's test for equality of variances, and thus Welch's *t* test used. **p < .01.

**Figure 1.**
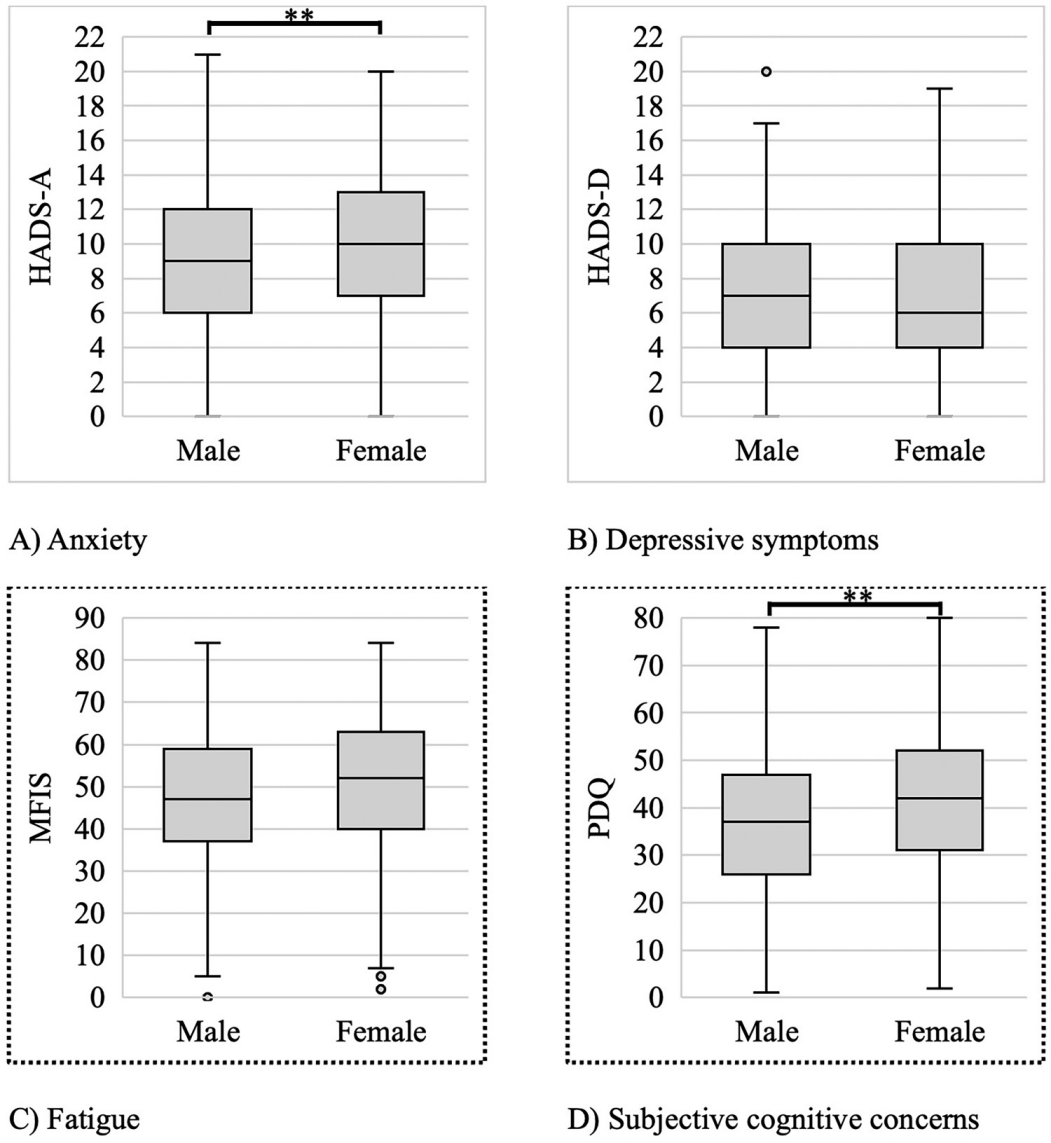
Sex differences in anxiety, depressive symptoms, subjective cognitive concerns, and fatigue. (A) Hospital Anxiety and Depression Scale sub-scale for Anxiety (HADS-A), (B) Hospital Anxiety and Depression Scale sub-scale for Depression (HADS-D), (C) Modified Fatigue Impact Scale (MFIS), and (D) Perceived Deficits Questionnaire (PDQ). *t*-tests show significant (** if *p* < .01) differences between males and females. In each box and whisker plot, the center line denotes the median, the box contains the interquartile range, the whiskers extend to 1.5 times the interquartile range, and the circles denote outliers. Dots surrounding exterior of graph indicate significant relationship between variable and sex after adjusting for covariates.

**Figure 2.**
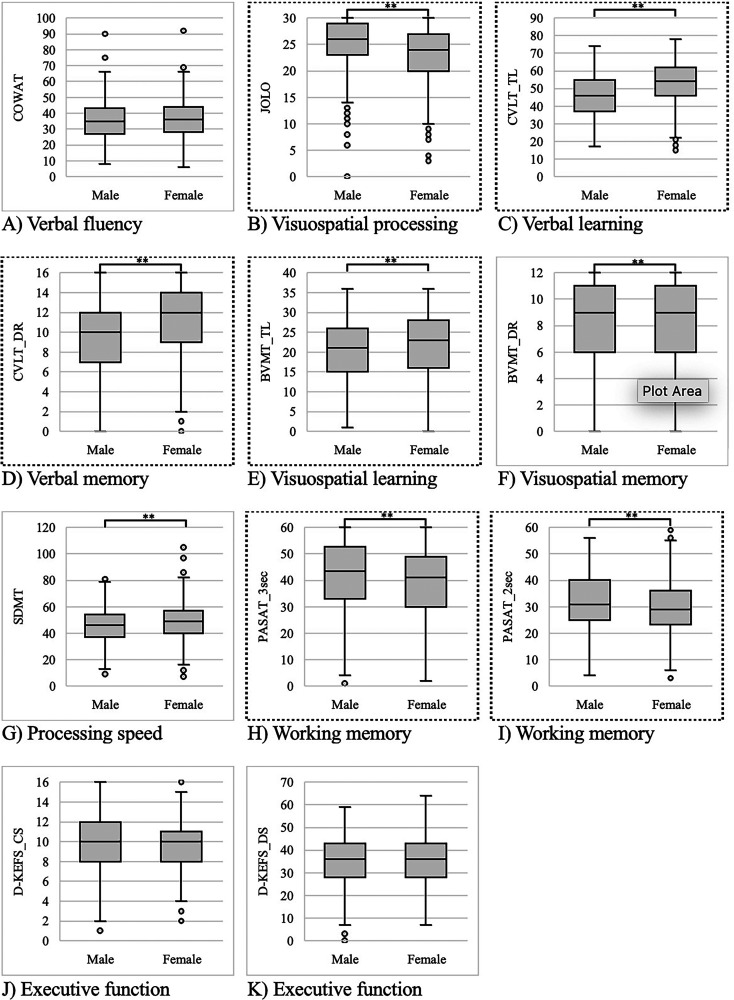
Sex differences in the minimal assessment of cognitive function in multiple sclerosis (MACFIMS) neuropsychological battery. (A) Controlled Oral Word Association Test, (B) Judgment of Line Orientation test, (C) California Verbal Learning Test Second Edition (CVLT) total learning (CVLT_TL), (D) CVLT descriptive score (CVLT_DS), (E) Brief Visuospatial Learning Test – Revised (BVMT) total learning (BVMT_TL), (F) BVMT delayed recall (BVMT_DR), (G) Symbol Digit Modalities Test (SDMT), (H) Paced Auditory Serial Addition Test (PASAT) 3-s version (PASAT_3sec), (I) PASAT 2-s version (PASAT_2sec), (J) Delis-Kaplan Executive Function System (D-KEFS) correct sorts (D-KEFS_CS), and (K) D-KEFS descriptive score (D-KEFS_DS). *t*-tests show significant (** if *p* < .01) differences between males and females. In each box and whisker plot, the center line denotes the median, the box contains the interquartile range, the whiskers extend to 1.5 times the interquartile range, and the circles denote outliers. Dots surrounding exterior of graph indicate significant relationship (*p* < .01) between variable and sex after adjusting for covariates.

As noted in Table 2, females performed better than males on the CVLT (both tests), BVMT (both tests), and SDMT, all *p* < .01. In contrast, males had better scores than females on the JOLO and PASAT (both tests), all *p* < .01. In addition, females had higher HADS-A and PDQ scores than males, both *p* < .01. There were no significant sex differences in HADS-D, MFIS, COWAT, D-KEFS_CS, or D-KEFS_DS scores. Box and whisker plots of neuropsychiatric symptoms stratified by sex are illustrated in Figures 1 and 2. Overlap coefficients illustrated a high degree of similarity between males and females across all neuropsychiatric sequelae of MS (0.94–1.00).

Adjusted for covariates, females had higher scores than males on the CVLT (both tests), and BVMT_TL, while males were better on the JOLO and PASAT (both tests), all *p* < .01. After controlling for covariates, females had higher MFIS and PDQ scores than males (both *p* < .01). Sex was not an independent predictor of scores on the HADS-A, HADS-D, COWAT, BVMT_DR, SDMT, D-KEFS_CS, or D-KEFS_DS. Table 3 describes standardized beta coefficients from these linear regression analyses.

**Table 3.** Linear Regression Models to Predict the Neuropsychiatric Sequelae of Multiple Sclerosis From Sex, Adjusted for Age, Years of Education, Neurological Disability, Disease Duration, and Disease Subtype.

	Sex	Age	Educational Years	EDSS	Disease Duration	Disease Subtype
HADS-A	.05	−.15**	−.03	−.05	.05	−.10**
HADS-D	−.04	−.05	−.08**	.15**	.02	−.08
MFIS	.10**	.02	−.17**	.19**	.03	−.08
PDQ	.09**	−.10**	−.15**	.05	.06	−.12**
COWAT	.03	.07	.19**	−.23**	−.02	.02
JOLO	−.22**	−.07	.11**	−.21**	−.05	.08
CVLT_TL	.20**	−.10**	.24**	−.28**	−.08**	.01
CVLT_DR	.16**	−.09**	.23**	−.29**	−.09**	.03
BVMT_TL	.07**	−.21**	.11**	−.23**	−.10**	.05
BVMT_DR	.06	−.19**	.10**	−.25**	−.09**	.08
SDMT	.06	−.16**	.11**	−.34**	−.07	−.01
PASAT_3sec	−.13**	.01	.16**	−.23**	.00	.01
PASAT_2sec	−.12**	.00	.19**	−.19**	.00	−.01
D-KEFS_CS	−.05	−.13**	.26**	−.27**	−.04	.05
D-KEFS_DS	−.03	−.10**	.25**	−.28**	−.03	.06

*Note:* EDSS = Expanded Disability Status Scale; HADS-A = Hospital Anxiety and Depression Scale sub-scale for symptoms of anxiety and depression; MFIS = Modified Fatigue Impact Scale; PDQ = Perceived Deficits Questionnaire; MACFIMS = Minimal Assessment of Cognitive Function in Multiple Sclerosis; COWAT = Controlled Oral Word Association Test; JOLO = Judgment of Line Orientation; CVLT_TL = California Verbal Learning Test Second Edition Total Learning score; CVLT_DR = California Verbal Learning Test Second Edition Delayed Recall score; BVMT_TL = Brief Visuospatial Memory Test Revised Total Learning score; BVMT_DR = Brief Visuospatial Memory Test Revised Delayed Recall score; SDMT = Symbol Digit Modalities Test; PASAT_3sec = Paced Auditory Serial Addition Test 3-s version; PASAT_2sec = Paced Auditory Serial Addition Test 2-s version; D-KEFS_CS = Delis-Kaplan Executive Function System Correct Sorts score; D-KEFS_DS = Delis-Kaplan Executive Function System Descriptive Score.

**p < .01.

## Discussion

In a large consecutive cohort of pwMS, this study demonstrates that generally, males exhibit greater dysfunction than females on tests of learning/memory and processing speed, while females perform worse on tests of visuospatial processing and working memory. After adjusting for demographic and disease-related factors, sex remains an independent predictor of verbal learning/memory, visuospatial learning, visuospatial processing, and working memory. While females experience more anxiety and subjective cognitive concerns compared to males, after controlling for covariates, sex independently predicts fatigue and subjective cognitive concerns, but not symptoms of depression or anxiety. The null results for depression and anxiety contrast with well-described sex differences in the general population.^25,26^

In interpreting these neuropsychiatric differences, the broader context of sex differences in MS is relevant. Females are more likely to develop MS and often exhibit heightened inflammatory activity compared to males.^1^ Yet, males experience greater disability progression,^1^ are more likely to have a progressive course of illness,^1^ and have elevated whole brain and gray matter atrophy in the thalamus and putamen compared to females.^1,2,14^

### Sex Differences in Cognition

Based on these clinical and radiological sex differences in MS, one may expect a broadly elevated burden of cognitive dysfunction in males with MS; however, the existing evidence is equivocal.^2,6,8,14–17^ There is also conflicting data about sex differences in the neuroradiological correlates of cognition in MS. While some studies suggest that total gray matter and thalamic atrophy may be more closely associated with cognitive dysfunction in males compared to females,^15,17^ other studies have not found a sex difference in the relationships between gray matter atrophy and cognition in pwMS.^8,14^ Regarding white matter, some studies demonstrate closer relationships between white matter abnormalities and cognitive dysfunction in males compared to females.^15,17^ In contrast, a separate study suggests heightened white matter disconnections in females with MS.^14^ A recent large study did not demonstrate any sex differences in the associations between multiple cognitive domains and brain parenchymal fraction or T2 lesion volume.^8^ In sum, prior to our study, data in pwMS were mixed about sex differences in cognition or sex-specific radiological correlates of cognition.

Our data clarify the pattern of cognitive sex differences in pwMS. Males experience elevated dysfunction in verbal learning/memory compared to females, in keeping with prior studies in pwMS.^6,8,14,15^ Males also perform worse in visuospatial learning, while females have mildly elevated difficulties in working memory. These data align with trends in some studies of pwMS,^14^ but not all.^6,15^ Both null studies were likely underpowered to detect sex differences.^6,15^ A recent study also found similar trends (not reaching significance) of higher BVMT_TL and lower PASAT scores in females compared to males.^8^ Finally, we find that females perform worse than males on a test of spatial rotation, a novel finding in pwMS, consistent with data from the general population.^27^ Dysfunction in visuospatial processing affects ∼20% pwMS,^21^ and may worsen at later times of day.^28^ It remains unknown whether this difference between males and females translates into functional differences in tasks that involve navigation or spatial rotation. Notably, these sex differences were independent of age, sex, years of education, neurological disability, disease duration, and illness subtype.

Although males have slower processing speed than females, our data show that this trend is not independent of demographic and disease-related factors. Most prior studies in MS have similarly not found a difference between males and females in processing speed.^2,6,14–16^ In a previous study that demonstrated higher processing speed in females than males, the investigators did not adjust for these potential confounding factors (e.g., age, sex, years of education, neurological disability, disease duration, or illness subtype).^17^ Nevertheless, even after controlling for demographic and disease-related variables, a large cohort showed a slight female advantage in processing speed.^8^ Compared to the present study, this cohort was slightly younger, showed less cognitive dysfunction across all measures, and was and more likely to receive disease-modifying treatment. It is unclear whether these factors may account for the discrepant findings, meriting further study. Finally, the absence of a sex difference in executive function aligns with a prior study in pwMS.^6^ Although one study found mildly better executive function in females compared to males, the study was limited by its small sample size and no correction for clinical disease-related factors in the final regression model.^15^

Of note, the cognitive sex differences in pwMS resemble sex differences observed in the general population.^27,29^ In the general population, females have better verbal learning/memory, while males have stronger spatial processing and possibly arithmetic skills.^27^ Similar to the MS literature, there is mixed evidence about sex differences in visual learning/memory, processing speed, and executive function in the general population.^27,29^ While aware of a high degree of overlap between males and females with regard to cognition, our data elucidate sex differences in learning/memory, processing speed, working memory, and visuospatial processing in pwMS, a starting point to clarify distinct radiological correlates.

### Sex Differences in Subjective Cognitive Concerns

In contrast to findings about objective cognition, we demonstrated that females with MS experience greater cognitive concerns than males with MS even after controlling for differences in age, years of education, and several disease-related factors. Although this result aligns with a recent large study,^8^ it differs from a prior investigation.^13^ Failures to account for the influences of neurological disability and disease duration may contribute to divergent findings.^13^ In the general population, recent evidence has not demonstrated a difference in the severity of subjective cognitive perceptions between males and females,^30^ suggesting that this may be MS-specific.

It is unclear how to conceptualize subjective cognitive concerns in pwMS. There is a weak, inconsistent relationship between self-reported and performance-based measures of cognition.^31^ Posited explanations of this discrepancy include the poor sensitivity of assessment tools, limited insight into one's cognition, variability depending on the cognitive domain assessed, and the influence of other symptoms such as depression, anxiety, and fatigue.^31^ Links between heightened subjective cognitive concerns and reduced volume of the cortex,^32^ thalamus,^32^ and hippocampus^33^ support neuro-anatomical correlates of these concerns, but do not resolve competing hypotheses.

Adding to this debate, concomitant with elevated subjective cognitive concerns in females, we observed increased fatigue, and dysfunction in working memory and spatial rotation in females with MS, but no sex differences in depression and anxiety. In the general population, there are sex differences in the relationships between self-reported concerns and performance-based cognitive measures and hippocampal atrophy.^34,35^ However, these questions have not been examined in pwMS. Subjective cognitive concerns are distressing for pwMS and elevated self-reported concerns in females suggest the need to explore how sex may influence the relative importance of potential contributors to these concerns.

### Sex Differences in Fatigue

Although there was a trend toward greater fatigue in females than males in the raw data, this relationship was only unmasked once we accounted for education and neurological disability. In pwMS, most studies demonstrate elevated fatigue in females compared to males,^10,11^ a sex difference that is also seen in the general population.^36^ Although the North American Research Committee on Multiple Sclerosis survey suggested more severe fatigue in males compared to females, this analysis did not account for the influences of neurological disability and education.^12^ It was necessary to adjust for these variables to uncover the true relationship between sex and fatigue. Potential contributors to MS fatigue include cortico-striato-thalamo-cortical loop abnormalities, inflammation, depression, anxiety, and sleep disorders.^37^ It is thus again intriguing that we found no sex difference in symptoms of depression or anxiety. Further sex-specific study is needed to ensure that fatigue is appropriately addressed for all pwMS.

### Depressive Symptoms in MS

Notably, there was no difference in depressive symptom severity between males and females, aligning with the majority of studies to examine this question.^6,7^ While depression may be more frequently diagnosed in females compared to males,^4^ this may reflect under-recognition of depression in males with MS. As a result, diagnostic data (in contrast to patient-reported outcomes) may contribute to an inaccurate understanding of sex differences in depression. Another study that demonstrated a sex difference in depressive symptoms used the Beck Depression Inventory (second edition).^9^ However, this scale includes questions about somatic symptoms, which are higher in females than males with MS.^9^ We similarly found a greater burden of fatigue in females compared to males. When the investigators only examined the cognitive symptoms of depression, there was no sex difference in depressive symptom severity.^9^ By evaluating depressive symptoms using the HADS,^22^ a patient-reported outcome measure that excludes most of these somatic symptoms, we did not demonstrate a sex difference in depressive symptoms in pwMS.

This repeated null finding is in striking contrast to the 2:1 ratio of depression between females and males in the general population.^25^ Studies in the general population consider that these sex differences may relate to differences in sex hormones, coping styles, personality, trauma and violence, and sociocultural factors.^25^ It remains unclear why these factors do not result in a similar distribution of depressive symptoms in pwMS. Some research in the general population suggests that the HADS subscale for depression may mitigate sex differences in depressive symptom severity.^38^ Yet, studies using other measures of depression have also not found a sex difference in pwMS.^7^ As another explanation, it is possible that disease-specific factors are particularly influential in contributing to depression risk in pwMS. For example, MS lesions to a transdiagnostic neural circuit rooted in the ventral tegmental area, reduced deep gray matter volume, or cerebellar atrophy may all contribute to depressive symptoms in pwMS.^39–41^ However, none of these studies assessed for sex differences. Alternatively, it is possible that sex-specific factors account for a high prevalence of depressive symptoms in males with MS. Yet, few studies focus on depressive symptoms in males with MS – a potential avenue for research.

### Anxiety in MS

Similarly, studies in pwMS fail to find a sex difference in anxiety symptoms.^6,7^ Those that describe elevated anxiety in females with MS are limited by the use of diagnoses rather than symptom severity,^4^ or not adjusting for confounding variables.^5^ In our unadjusted sample, we also found higher anxiety in females compared to males; however, by controlling for demographic and disease-related variables, it becomes evident that the severity of anxiety is similar between males and females with MS. In the general population, akin to depression, there is a higher prevalence of anxiety in females than males,^26^ with similar mechanisms thought to explain this sex difference.^26^ The absence of a sex difference in anxiety in pwMS emphasizes that factors unrelated to sex may heavily contribute to anxiety risk in MS.

## Conclusions

It is also relevant to consider how these findings relate to theories of reserve in pwMS. In the context of disease activity and progression, pwMS exhibit variable cognitive and affective outcomes. Models of cognitive and affective reserve hypothesize that life activities (e.g., education and work) or psychosocial characteristics (e.g., personality, coping styles, and resilience) respectively may buffer against the development of cognitive dysfunction or depression in the face of disease-related adversity.^42,43^ The only study to date that investigated this potential interaction revealed no evidence one on neurocognitive outcomes.^42^ Although there are sex differences in personality traits in the general population,^44^ it remains unknown whether there are differences in affective reserve between males and females with MS. Building upon our investigation, future studies should examine whether sex differences in cognitive or affective reserve impact neuropsychiatric outcomes.

Potential study limitations include a bias toward relapsing illness, and no data on neuroimaging, hormonal levels, or psychotropic medication use. In addition, there is no healthy control group and the only published Canadian MACFIMS normative dataset adjusts for sex, but does not report sex-specific normative data.^45^ These factors limit conclusions about whether differences in cognitive performance are specific to MS and emphasize the need for publication of sex-stratified Canadian normative data for all the included neuropsychiatric measures. Nonetheless, this study reveals that among pwMS, on average, males perform worse than females on verbal learning/memory and visuospatial learning tests, while females exhibit reduced scores on measures of working memory and spatial rotation. Although females experience greater fatigue and subjective cognitive concerns than males, there are no sex differences in the severity of depression or anxiety, in contrast to a female predominance of these symptoms in the general population. These data expand our understanding of neuropsychiatric sex differences in pwMS in clinical practice, and create a foundation for the future research that can explain sex-related mechanisms of neuropsychiatric symptoms in MS.
